# Cytoplasmic dynein and its regulatory proteins in Golgi pathology in nervous system disorders

**DOI:** 10.3389/fnins.2015.00397

**Published:** 2015-10-26

**Authors:** Dick Jaarsma, Casper C. Hoogenraad

**Affiliations:** ^1^Department of Neuroscience, Erasmus MCRotterdam, Netherlands; ^2^Cell Biology, Faculty of Science, Utrecht UniversityUtrecht, Netherlands

**Keywords:** Golgi, dynein, dynactin, BICD2, Golgin 160, Lis1, transport, neurodegenerative disorders

## Abstract

The Golgi apparatus is a dynamic organelle involved in processing and sorting of lipids and proteins. In neurons, the Golgi apparatus is important for the development of axons and dendrites and maintenance of their highly complex polarized morphology. The motor protein complex cytoplasmic dynein has an important role in Golgi apparatus positioning and function. Together, with dynactin and other regulatory factors it drives microtubule minus-end directed motility of Golgi membranes. Inhibition of dynein results in fragmentation and dispersion of the Golgi ribbon in the neuronal cell body, resembling the Golgi abnormalities observed in some neurodegenerative disorders, in particular motor neuron diseases. Mutations in dynein and its regulatory factors, including the dynactin subunit p150Glued, BICD2 and Lis-1, are associated with several human nervous system disorders, including cortical malformation and motor neuropathy. Here we review the role of dynein and its regulatory factors in Golgi function and positioning, and the potential role of dynein malfunction in causing Golgi apparatus abnormalities in nervous system disorders.

## Introduction

**Figure 1:**
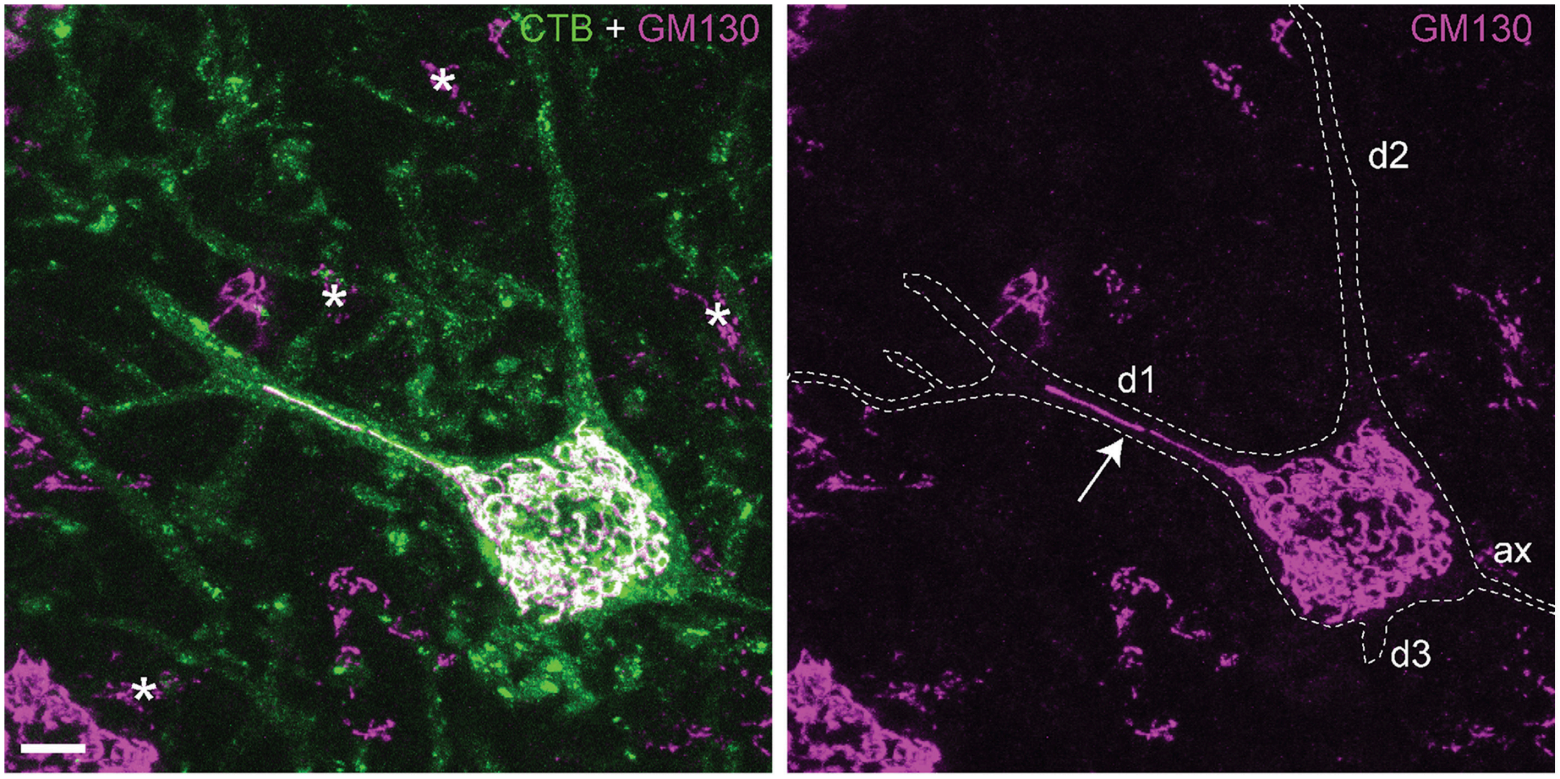
**Somatic and dendritic distribution of Golgi apparatus in spinal motor neurons**. Projection of confocal sections (optical thickness = 15 μm) of a retrogradely Cholera toxin B (CTB) labeled motor neuron double labeled for cis-Golgi matrix protein GM130 (van Dis et al., 2014). CTB outlines the somato-dendritic compartment of labeled motor neurons, accumulating in the cytoplasm, trans-Golgi and lysosomes. GM130 immunoreactivity is associated with an extensive perinuclear network of thread-like profiles, that in motor neurons may enter into the initial part of one or more dendrites. This example shows one GM130-positive profile extending in the center of the dendrite up to the first branching point (arrow), while other dendrites (d2, d3) do not show Golgi profiles. In other neurons the Golgi profiles may extend up to the second branching point, or multiple profiles running parallel may invade a single dendrite (Gardiol et al., 1999; van Dis et al., 2014). Asterisks, Golgi apparatus of glia cells; Bar = 10 μm.

The Golgi apparatus consists of stacks of biochemically and functionally heterogeneous disk-shaped cisternae. In vertebrate cells, the Golgi stacks are laterally connected to form a single continuous membrane system, termed the Golgi ribbon that usually localizes near the centrosome (Klumperman, 2011). Golgi ribbon size and morphology may vary between cell types according to specific requirements of the secretory pathway (Yadav and Linstedt, 2011; Nakamura et al., 2012; Watanabe et al., 2014). In neurons the Golgi ribbon forms an extensive perinuclear network that in multiple neuron types may extend into one or more dendrites (Figure 1) (Gardiol et al., 1999; Hanus and Schuman, 2013), and is complemented by Golgi fragments in distal dendrites, designated Golgi outposts (Horton et al., 2005; Ori-McKenney et al., 2012; Quassollo et al., 2015).

The biogenesis and maintenance of the Golgi ribbon structure and position strongly depends on the microtubule cytoskeleton and microtubule motors, in particular cytoplasmic dynein that drives microtubule minus-end transport. Microtubule depolymerization with nocodazole results in the fragmentation of the Golgi ribbon, and the rebuilding of secretion competent ministacks dispersed throughout the cell at endoplasmic reticulum (ER) exit sites. The ministacks are reminiscent of the Golgi apparatus organization found in non-vertebrate organisms, including plants and Drosophila (Cole et al., 1996; Kondylis and Rabouille, 2009). While radial microtubules emanating from the centrosomal microtubule-organizing center mediate pericentrosomal positioning of the Golgi, the vertebrate Golgi apparatus also is a microtubule-organizing center by itself. Golgi-nucleated microtubules, among other functions, are important for assembly of the Golgi ribbon (Zhu and Kaverina, 2013; Rios, 2014).

Also inhibition of cytoplasmic dynein function results in fragmentation of the Golgi ribbon into dispersed, secretion-competent, Golgi ministacks (Palmer et al., 2009; Yadav and Linstedt, 2011). In the Golgi apparatus, cytoplasmic dynein drives the bulk of afferent transport of membrane cargo, i.e., transport from the ER to the cis (entry)-side of the Golgi, and retrograde transport from post-Golgi compartments (endosomes, lysosomes) to the trans (exit)-side of the Golgi (Yadav and Linstedt, 2011; Stephens, 2012; Lord et al., 2013; Lu and Hong, 2014). To accommodate its many functions, a repertoire of factors regulates its subcellular recruitment, cargo interactions, and its mechano-chemical properties (Akhmanova and Hammer, 2010; Fu and Holzbaur, 2014; Jha and Surrey, 2015). The uncovering of Golgin160 as an important dynein adaptor for Golgi membranes has provided basic insight into how dynein may maintain Golgi ribbon structure and position (Yadav et al., 2012). However, knowledge about the role of dynein in the coordinated trafficking of pre-, intra, and post-Golgi membranes, and how mutations in dynein and its regulators affect Golgi function, is still very limited. Here we review the role of dynein and its accessory factors in Golgi membrane trafficking. In addition, since mutations in dynein and its regulators are linked to a variety of nervous system disorders, we will discuss the possible link between disease associated mutations and Golgi abnormalities.

## Cytoplasmic dynein motor—dynein heavy chain

**Figure 2:**
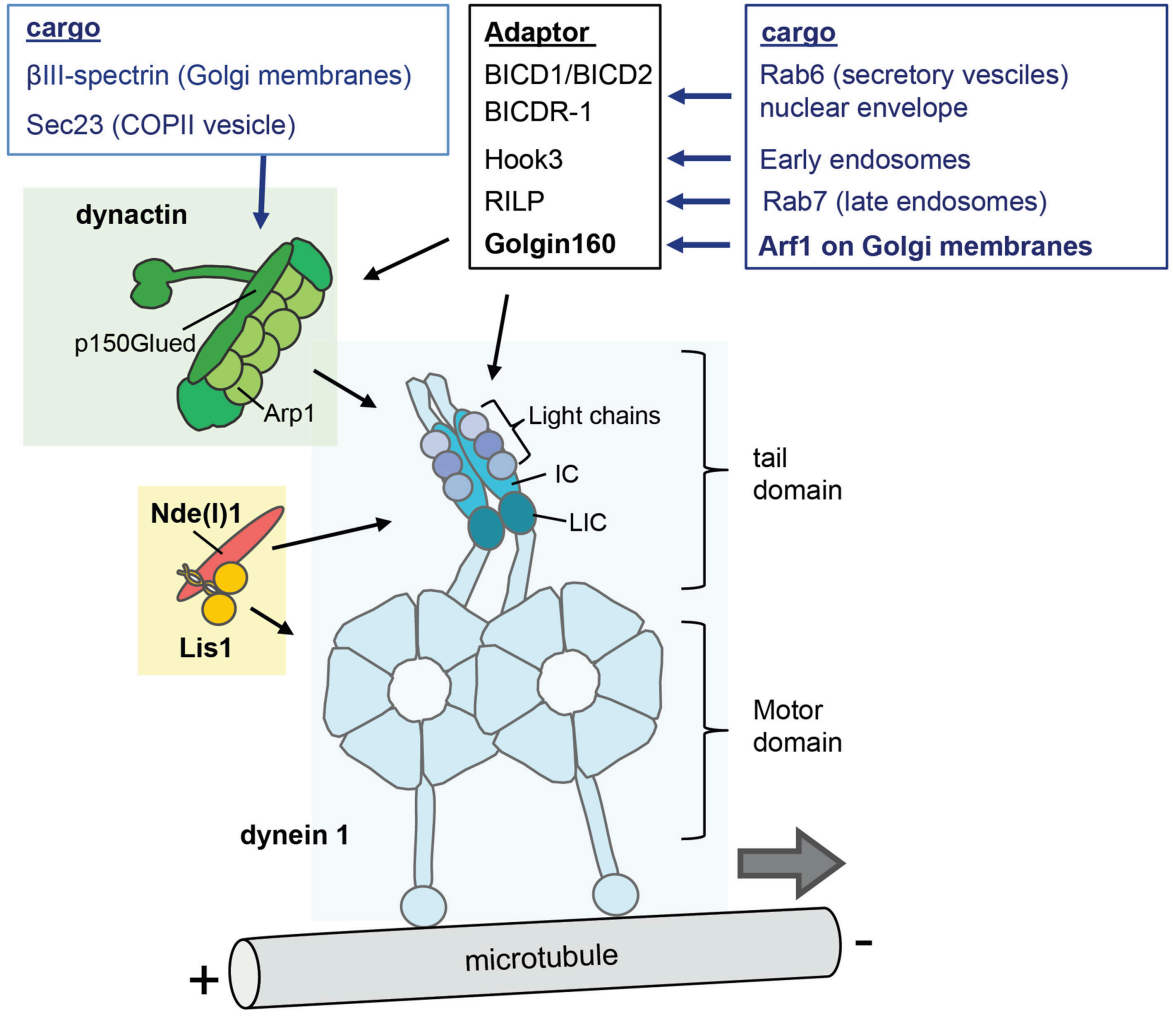
**The cytoplasmic dynein complex and its regulators**. The dynein 1 molecule is a complex of two heavy chains (HC), two intermediate chains (IC), two light intermediate chains (LIC), and three light chains. The motor domain comprises six AAA+ ATPase modules that form a ring and a stalk that contains the microtubule domain. The N-terminal tail domain interacts with the smaller non-catalytic subunits, and is responsible for dimerization and cargo interaction. The dynactin complex (green) and Lis1/Nde(l)1 (orange/red) are key factors in the regulation of cargo interaction and motor function. Dynactin contains a filament of eight copies of the actin-related protein Arp1, the large subunit p150glued, and 14 additional subunits including one copy of β-actin, Arp11 and p50 dynamitin (Urnavicius et al., 2015). In addition, a few dynein cargo-adaptors are indicated that regulate the interactions with specific cargo's.

Cytoplasmic dynein 1 (hereafter dynein) is a two-headed microtubule motor that is used for nearly all of the minus-end directed transport, the other dyneins operating in cilia and flagella to power ciliary and flagellar beating (axonemal dyneins) or intraflagellar transport (dynein 2) (Vallee et al., 2012; Carter, 2013; Roberts et al., 2013). Dynein consists of a dimer of the motor-containing heavy chain (DYNHC1H1) and several smaller accessory subunits (Figure 2). The motor domain of DYNHC1H1 is in the C-terminus and consists of a ring of six AAA+ ATPase modules, and a 15 nm stalk domain which is responsible for microtubule binding and generating movement along the microtubules (Vallee et al., 2012; Carter, 2013; Roberts et al., 2013). The N-terminal tail domain is responsible for dimerization and cargo interaction.

Dynein heavy chain knockout mice die before implantation and show dispersed Golgi in early embryonic cells (Harada et al., 1998). Heterozygous DYNC1H1 mutations are associated with a spectrum of nervous system abnormalities, with, roughly, tail domain mutations causing a form of spinal muscular atrophy (SMA) or motor-sensory neuronopathies, motor domain mutations causing cortical malformation, and some mutations causing combined phenotypes (Lipka et al., 2013; Peeters et al., 2015; Scoto et al., 2015). These data indicate that mutations may differentially affect the many cellular dynein functions. A tail mutation causing a motor neuronopathy in mice (*Loa* mice) has been shown to reduce dynein processivity, and to cause impaired retrograde axonal transport, as well as delayed Golgi ribbon reassembly following nocodazole washout (Hafezparast et al., 2003; Vallee et al., 2012). Also mutations in the tail and motor domains associated with combined SMA/cortical malformation cause delayed Golgi ribbon reassembly following nocodazole washout, suggesting impaired Golgi membrane trafficking in many DYNC1H1 patients (Fiorillo et al., 2014). Recently, some dynein tail-mutations (R264L, R598) causing SMA have been found to cause increased binding to the cargo-adaptor BICD2 (see below) (Peeters et al., 2015). Significantly, mutations in BICD2 cause similar motor neuron phenotypes, and are also associated with Golgi abnormalities (Neveling et al., 2013; Peeters et al., 2013).

## Cytoplasmic dynein motor—dynein accessory subunits

The dynein smaller accessory subunits also occur in two copies each, and comprise the intermediate chains (IC), the light-intermediate chains (LIC) and three classes of light chains (Figure 2). In primates and rodent two genes encode each of the smaller subunits, while additional diversity is generated by alternative splicing of IC, and phosphorylation of IC and LIC isoforms (Pfister et al., 2006; Allan, 2011). The IC acts as a scaffold between the heavy chains and the light chains and is a major binding platform for dynein interacting proteins including dynactin. Of the two IC isoforms, IC1 and IC2, IC2 is expressed in all cells and is essential for all dynein functions in non-neuronal cells including Golgi apparatus function and positioning (Pfister et al., 2006; Palmer et al., 2009; Raaijmakers et al., 2013). IC1 is predominantly expressed in neurons, with different roles for the various IC1 and IC2 isoforms in axonal transport (Pfister, 2015).

The LIC dimer directly attaches to dynein heavy chain with a Ras-like domain, and can bind cargo adapter proteins such as FIP3, RILP, and BICD2 with its C-terminus (Schroeder et al., 2014). The two LIC subtypes, LIC1 and LIC2, assemble as homodimer into two different populations of dynein. “LIC1-dynein” and “LIC2-dynein” have been implicated in different functions, but the extent to which they are functionally redundant is uncertain (Allan, 2011; Raaijmakers et al., 2013). A knockdown study suggested specific LIC1 functions in ER-Golgi trafficking (Palmer et al., 2009; Brown et al., 2014), but no Golgi abnormalities were found after LIC1 knockdown by others (Allan, 2011; Tan et al., 2011). Mice, derived from a mutagenesis screen, that were homozygous for a missense mutation (N215Y) in LIC1, and showed mild behavioral changes combined with changes in dendritic morphology also showed abnormalities in Golgi ribbon reassembly following exposure to nocodazole, pointing to a role of LIC1 in Golgi membrane trafficking (Banks et al., 2011). However, LIC1 knockout mice display normal Golgi apparatus, although some evidence suggests a specific role of LIC1 in ER export (Kong et al., 2013). LIC1 knockout mice also show photoreceptor degeneration resulting from impaired dynein dependent transport of Rab11-vesicle trafficking from the Golgi apparatus to the base of the connecting cilium (Kong et al., 2013).

Dynein light chains consists of LL1/2 (also known as LC8-1/2), Roadblock-1/2, and LT1/3 (also known as TCTex1 and TCTex1L or rp3, respectively) that associate with dynein via specific binding sites on the intermediate chains (Pfister et al., 2006; Allan, 2011). The light chains are involved in regulating dynein complex assembly and cargo interactions but in addition may have functions independent of dynein, in particular LL1. Knockdown of LT1 and Roadblock-1 was found to cause Golgi dispersion, while knockdown of LL1 may result in subtle changes in ER to Golgi transport, and LT3 (rp3) knockdown did not alter Golgi markers (Palmer et al., 2009). Another study used rapamycin-inducible ligands that upon dimerization act as molecular traps for LT1 and LL1, respectively (Varma et al., 2008). Both LT1 and LL1 traps induced Golgi dispersion after induction of dimerization. However, despite relatively rapid induction of light-chain inactivation, and, accordingly a dispersion of lysosomes and endosomes within 1 h after light chain trap activation, Golgi dispersion was much slower, suggesting that the effect on Golgi fragmentation is indirect (Varma et al., 2008).

## Regulators of dynein activity—dynactin

Dynactin is a 1 MDalton multiprotein complex of more than 20 subunits that interacts with the dynein tail domain and mediates dynein-cargo interaction, recruits dynein at microtubule plus-ends, and is required for processive movement (i.e., the ability to make multiple steps before releasing the microtubule) of dynein (Jha and Surrey, 2015; Urnavicius et al., 2015). Major dynactin components include a filament of actin-related protein 1 (Arp1) and a large subunit, p150glued (also designated DCTN1) that mediate dynein interaction (Figure 2) (Urnavicius et al., 2015). P150glued can bind microtubules by itself via its N-terminus that contains a conserved CAP-Gly domain. This domain is dispensable for activation of dynein processivity, but is required for other functions such as recruitment of dynein to microtubule plus-ends (Akhmanova and Hammer, 2010; Fu and Holzbaur, 2014; Jha and Surrey, 2015).

Heterozygous point mutations in the p150glued N-terminus cause rare adult onset neurodegenerative disorders (Lipka et al., 2013). Mutations associated with a Parkinsonian disorder (Perry syndrome) afflict microtubule binding and may cause the accumulation of cargo in axon terminals, which may be explained by impaired recruitment of dynactin at microtubule plus-ends in axon terminals (Lloyd et al., 2012; Moughamian et al., 2013; Tacik et al., 2014). Instead a mutation (G59S) associated with a motor neuron disease, is thought to afflict p150Glued stability and to more generally impair dynactin-dynein functions (Lipka et al., 2013). This mutation also may interfere with Golgi apparatus homeostasis, as patient-derived cells show retarded recovery of Golgi after nocodazole treatment (Levy et al., 2006).

Disruption of dynactin or the dynactin/dynein interaction by overexpression of the p50 dynamitin subunit or a dominant p150Glued construct (cc1), respectively is well known to cause Golgi fragmentation and dispersion, and dynactin has been implicated in several steps in ER-to Golgi transport (Yadav and Linstedt, 2011; Lord et al., 2013). Dynactin can directly bind Golgi membranes via its Arp1 subunit that binds βIII spectrin on Golgi membranes (Yadav and Linstedt, 2011). βIII spectrin depletion has been found to cause Golgi fragmentation and impaired Golgi reassembly after nocadozole treatment, consistent with a role in dynein/dynactin function in the Golgi apparatus (Salcedo-Sicilia et al., 2013). Arp1-spectrin interaction also is implicated in dynein dependent transport of Golgi membranes in Drosophila during the cellularization of the larvae (Papoulas et al., 2005). Mutations in βIII-spectrin are associated with a neurodegenerative disease that affects the cerebellar Purkinje cells (SCA5), and βIII-spectrin-deficient mice or mice expressing SCA5-mutant βIII-spectrin develop Purkinje cell degeneration. However, so far there are no neuropathological data indicative of abnormalities in dynein-dependent motility of Golgi membranes in these mice (Armbrust et al., 2014; Clarkson et al., 2014).

Dynactin also can bind specific membrane compartments via its p150glued subunit. P150glued interacts with Sec23 of the COPII vesicle coat complex that sort cargo into budding vesicles at ER exit sites for their delivery at the juxtaposed tubulo-vesicular ER-Golgi intermediate compartment (ERGIC). Although disruption of this interaction slows-down ER exit, the dynactin-Sec23 binding is not essential for ER-ERGIC trafficking (Watson et al., 2005; Yadav and Linstedt, 2011; Lord et al., 2013). A suggested role for dynactin-Sec23 interaction is to aid in the separation of the nascent COPII vesicle (Bacia et al., 2011). Such a role for dynein/dynactin has been suggested in the formation of retromer-coated vesicles at endosomes. Here p150glued binds SNX6 of the SNX-BAR-retromer to first aid with separation of the retromer-coated cargo and then mediate transport from endosomes to the trans Golgi network (TGN) (Wassmer et al., 2009; Stephens, 2012). Interestingly, the interaction between p150glued and SNX6 is negatively regulated by phosphotidylinositol-4-phosphate, a Golgi-enriched phosphoinositide that strongly binds SNX6. Thus, at the TGN phosphotidylinositol-4-phosphate stimulates the dissociation of p150glued and SNX6, providing a mechanism of cargo release by dynein/dynactin (Niu et al., 2013).

## Regulators of dynein activity—Lis1, Nde1, and Ndel1

Lis1, Nde1 (nuclear distribution protein E, also known as NudE) and the paralogue Ndel1 are key dynein regulators that can alter its mechanochemical properties, and are involved in most if not all of its functions (Figure 2) (Vallee et al., 2012; Roberts et al., 2013). Lis1 is a 45 kD protein that as a dimer can bind the dynein heavy chain motor domain, and can promote a persistent microtubule bound state by uncoupling ATP hydrolysis cycles from microtubule binding affinity changes (Vallee et al., 2012; Toropova et al., 2014). Lis1 is implicated in dynein recruitment and regulation, and is thought to be especially important for high-load dynein functions, including initiation of axonal transport and high load functions in cell migration and mitosis (Yi et al., 2011; Splinter et al., 2012; Vallee et al., 2012; Raaijmakers et al., 2013).

Lis1-dynein interactions are modulated by Nde1 and Ndel1. Nde1 and Ndel1 are important for many dynein functions, either by regulating the recruitment of Lis1, regulating cargo interaction or via other mechanisms (Torisawa et al., 2011; Vallee et al., 2012). In the nervous system, Nde1 and Ndel1 show a largely complementary spatio-temporal expression in neuronal progenitors and post-mitotic neurons, respectively, indicative of different cellular functions, i.e., roles in neurogenesis vs. neuronal migration/morphogenesis, respectively (Sasaki et al., 2005; Pandey and Smith, 2011). The roles of Lis1 and Nde(l)1 are dosage dependent and their concentrations appear to be limiting for several dynein functions. Accordingly, patients and mice with one inactive Lis1 allele display defects in neuronal migration, while additional reduction of Lis1 or coincident reduction of Nde(l)1 results in more severe phenotypes (Sasaki et al., 2005; Youn et al., 2009).

The roles of Lis1 and Nde(l)1 in regulating dynein in the Golgi apparatus are not well understood. Lis1 or combined Nde1-Ndel1 knockdown result in Golgi fragmentation and dispersion in Hela cells, however some conflicting results have been reported in additional studies (Lam et al., 2010). Drosophila Nde1 was found to associate with Golgi membranes and to be required for dendritic targeting of Golgi outposts (Arthur et al., 2015). Notably, Lis1 also has a role as a structural subunit in a phospholipase A enzyme complex, termed PAFAHIb, which deacylates Golgi phospholipids and contributes to morphological remodeling of Golgi membranes. Some evidence indicate that catalytic PAFAHIb subunits compete with Ndel1 for Lis1 binding, and that Lis1 undergoes conformational changes when it switches from a complex with PAFAHIb subunits to one with Ndel1 (Ha et al., 2012).

## Activation of cytoplasmic dynein motility

**Figure 3:**
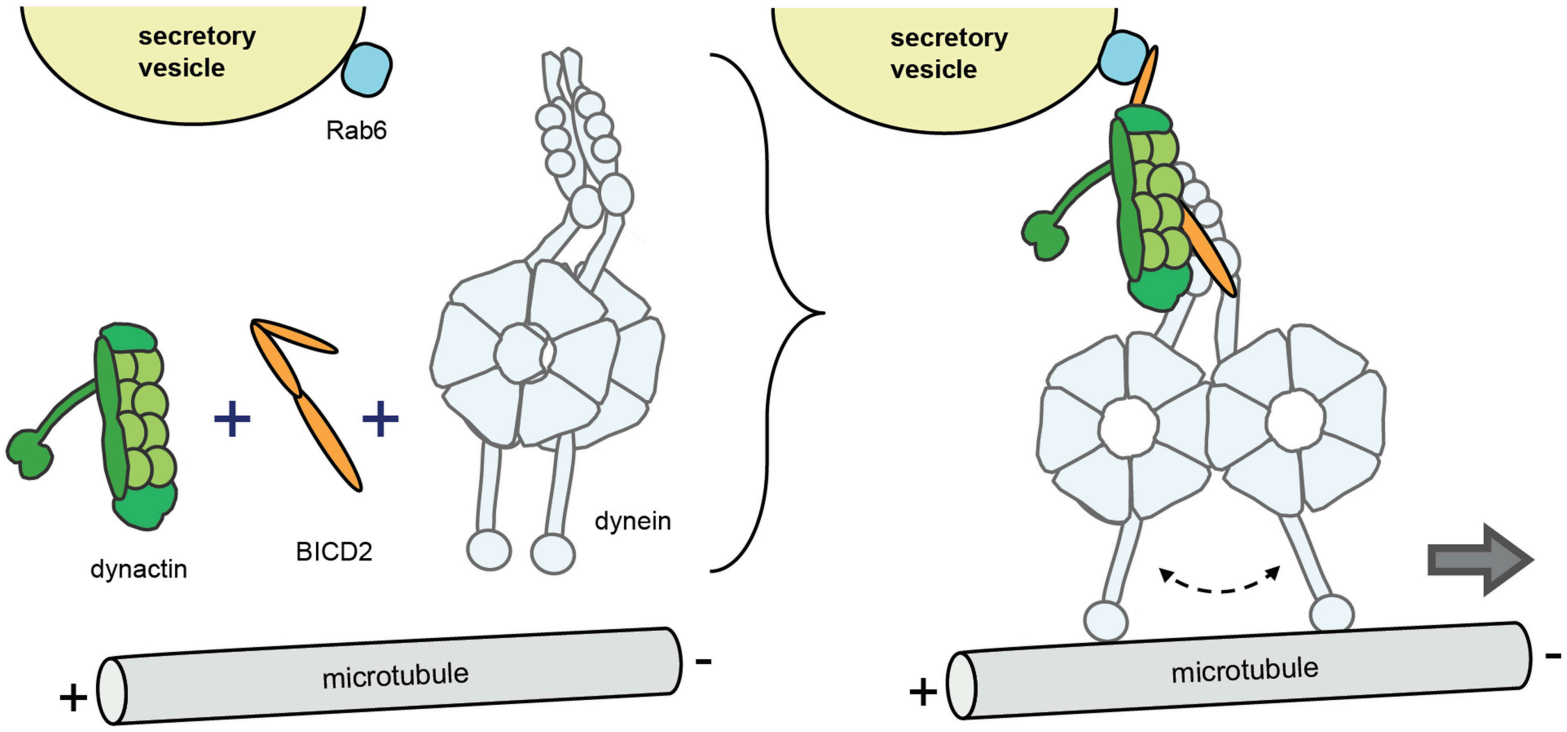
**Model of regulation of processive dynein/dynactin motility by cargo adaptors**. The model is based on findings with the conserved coiled coil protein BICD2 that operates as a dynein-dynactin cargo adaptor for a variety of cargo including Rab6 vesicles, nuclear envelope, and RNA particles (in Drosophila). Upon cargo binding (in this case Rab6 secretory vesicles), BICD2 co-recruits dynein and dynactin and forms a stable ternary complex to initiate processive minus-end movement. The model implies that dynein in the ternary complex experiences conformational changes in the motor domains, in particular the separation of the motor heads.

Although dynein subunits, dynactin and Lis1/Nde(l)1 regulate more general aspects of cargo interaction, an additional level of dynein control is provided by cargo-specific adaptor proteins (Akhmanova and Hammer, 2010; Vallee et al., 2012; Jha and Surrey, 2015). A group of these cargo-specific regulatory factors, including BICD1 and BICD2 (nuclear envelope, Rab6 vesicles), BICD-related protein 1 (Rab6 vesicles), Hook3 (early endosomes), Rab11-FIP3 (Rab11 recycling endosomes), RILP (Rab7 late endosomes/lysosome); TRAK1 and 2 (mitochondria), Spindly (kinetochores) and Golgin160 (Arf1 on Golgi membranes) contain coiled coils that bind dynein and/or dynactin (Figure 2). Recent studies showed that cargo specific adaptor proteins are also essential cofactors that can activate processive motor motility. Biochemical experiments found that mixing purified mammalian dynein and dynactin with the N-terminal fragment of BICD2 (BICD2-N) results in a stable ternary complex that mediates highly processive unidirectional movement. While dynactin alone is not sufficient for dynein activation, BICD2-N is required to link the two complexes together in order to initiate processive minus-end directed motility (Splinter et al., 2012; McKenney et al., 2014; Schlager et al., 2014a). Structural data show that the BICD2-N coiled coil extends between dynactin and the tail of dynein engaging in multiple reciprocal interactions (Urnavicius et al., 2015). Another study has shown that in inactive non-processive dynein, the two motor heads are stacked together, and that processive motility can be induced by separation of the motor heads (Torisawa et al., 2014). Together the data suggests a model where the binding of the cargo adaptor and dynactin induces conformational changes in the motor domains, promoting dissociation of motor heads and thereby activating directional motility (Urnavicius et al., 2015) (Figure 3). Interestingly, recent evidence indicates that different BICD cargo adaptors may differentially regulate speed and processivity of dynein (Schlager et al., 2014b). This can be explained by a mechanism where different adaptors trigger different conformational changes in the dynein motor domains. Thus, different dynein-dynactin cargo adaptors not only differ in cargo binding, but also may differentially modulate motor activity (Schlager et al., 2014b).

## Dynein regulatory factors for golgi membranes

Golgin160 has been identified as a dynein cargo adaptor for Golgi membranes (Yadav et al., 2012). Golgin 160 knock-down results in dissociation of dynein from Golgi membranes, fragmentation and dispersion of the Golgi ribbon, and impaired ER to Golgi trafficking of temperature sensitive viral cargo protein (VSVG-GFP). Golgin160 interacts with dynein via its coiled-coil cc7 domain. Like BICD2-N (Hoogenraad et al., 2003), GFP-tagged cc7-domain by itself accumulates at the centrosome, and when attached to mitochondria causes pericentrosomal clustering of mitochondria, indicating that this domain by itself is sufficient to trigger dynein processivity independent of the presence or type of cargo (Yadav et al., 2012). Thus, Golgin160 may coordinate cargo-binding with dynactin-dynein association and initiation of processive motility in a similar way as BICD2. Expression of high levels of BICD2-N or the early endosome cargo-adaptor Hook3 have been shown to cause Golgi dispersion (Hoogenraad et al., 2001; Walenta et al., 2001; Teuling et al., 2008). A possible explanation is that BICD2-N and hook3 can interfere with Golgin160 binding to dynein-dynactin. This would imply that misregulation of non-Golgi cargo adaptors can indirectly cause abnormalities in Golgi ribbon structure and position, and could explain Golgi abnormalities in cells from patients with BICD2 mutations (Neveling et al., 2013; Peeters et al., 2013). There is no evidence, so far, that wild type BICD2 plays a direct role in Golgi membrane trafficking (Yadav and Linstedt, 2011) and, accordingly, there are no obvious abnormalities in Golgi morphology in BICD2 knockout mice (Jaarsma et al., 2014).

Golgin160 attaches to Golgi membranes via binding of its N-terminus to Arf1, a small GTPase that plays a central role in recruiting factors at ERGIC and Golgi membranes, in particular COPI (Altan-Bonnet et al., 2004; Yadav et al., 2012). Thus, dynein recruitment to Golgi membranes is regulated by activation and deactivation of Arf1, coinciding with the recruitment of other Arf1 effectors and the “Golgification” of ERGIC membranes near the ER exit sites (Yadav et al., 2012). The regulation by GTPases is common to other cargo adaptors which operate as adaptors of Rab6 (BICD1/2, BICDR-1), Rab7 (RILP) and Rab11 (Fip3). Golgin160-Arf1 mediates not only inward motility of peripheral ERGIC membranes, but also clustering of Golgi membranes for assembly of the Golgi ribbon, and maintenance of the Golgi ribbon structure and position near the cell center (Nakamura et al., 2012; Yadav et al., 2012). Interestingly, during mitosis, Golgin160 disassociates from Golgi membranes, which contributes to Golgi disassembly into vesicular structures for proper partitioning between daughter cells (Nakamura et al., 2012; Yadav et al., 2012).

ZW10, an adaptor protein involved in dynactin and dynein recruitment to mitotic kinetochores, has been proposed to act as a linker between dynein/dynactin and Golgi membranes. Inhibition of ZW10 by knock-down, anti ZW10 antibody or dominant-negative overexpression causes Golgi fragmentation and dispersal, and reduced minus-end directed motility of Golgi membranes (Varma et al., 2006). However, other studies suggest that ZW10 has a function in the Golgi independent of dynein (Arasaki et al., 2007; Majeed et al., 2014).

Lava lamp is a large coiled-coil protein, identified in Drosophila, which binds α-Spectrin on Golgi membranes and may also act as a dynein/dynactin adaptor for Golgi membranes (Papoulas et al., 2005). In Drosophila, dynein-dependent motility of Golgi is not needed for maintenance of Golgi apparatus structure and position, as Golgi stacks in Drosophila are dispersed throughout the cytoplasm next to ER exit sites, reminiscent of the dispersed Golgi in mammalian cells after microtubule depolymerization or inhibition of dynein/dynactin (Kondylis and Rabouille, 2009). Instead, dynein is required for specialized processes such as cellularization of synctial embryo's (Papoulas et al., 2005) and the development of complex dendritic arbors in neurons (Ye et al., 2007). In neurons, lava lamp regulates dynein-dependent transport of Golgi outposts to distal dendrites. Inhibition of lava lamp results in a shift of Golgi outposts from distal to proximal dendrites and a concomitant distal to proximal shift in dendrite branching (Ye et al., 2007), and a similar redistribution of Golgi outpost and dendritic branches occur in Drosophila with mutations in IC and LIC dynein subunits and Lis1 (Zheng et al., 2008), as well as Nde1 knock-down (Arthur et al., 2015). Lava lamp knockdown also causes Golgi outposts to appear in axons and these axons start branching. Analysis of mutants of ER-to-Golgi trafficking, and laser damage of individual Golgi outposts further showed that Golgi outposts are particularly important for dendrite arborization in Drosophila (Ye et al., 2007). A recent study showed that the interaction between lava lamp and dynein at dendritic Golgi outposts is regulated by Leucine-rich repeat kinase (Lrrk), the Drosophila homolog of Parkinson's disease-associated Lrrk2 (Lin et al., 2015). Lrrk loss-of-function Drosophila mutants show increased anterograde movement of Golgi outposts and increased dendritic branching. Lrrk suppresses anterograde (proximo-distal) movement of Golgi outposts via phosphorylation of Lava lamp and inhibiting its interaction with dynein (Lin et al., 2015). The significance of the findings on dynein-dependent motility of Golgi outposts in Drosophila for mammalian neurons remains to be established. A mammalian homolog for lava lamp has not been identified (Munro, 2011), and the extent to which dynein is important for Golgi outpost positioning in hippocampal and cortical neurons is not yet known.

## Perspectives

The uncovering of dynein adaptor proteins and regulatory factors has provided basic insight into how dynein mediates Golgi membrane trafficking and maintains Golgi ribbon structure and position (Yadav and Linstedt, 2011; Yadav et al., 2012). Several important research questions remain to be addressed. Are there other adaptors in addition to Golgin160 implicated in dynein-dependent Golgi membrane motility in mammalian cells? For instance, do specialized adaptors operate in specialized Golgi compartments such as dendritic Golgi outposts? Does Golgin160 co-recruit dynein and dynactin in a similar way as BICD2 (Schlager et al., 2014a; Urnavicius et al., 2015)? How is Golgin160-Arf1 binding coordinated with dynein/dynactin recruitment? Analysis of Drosophila BICD showed that cargo binding triggers conformational changes that promote dynein/dynactin binding, thus coordinating cargo-binding with dynein recruitment (Liu et al., 2013). How do Lis1 and Nde(l)1 operate in Golgi membrane trafficking? Systematic siRNA-based dissection of dynein regulators in mitosis, indicated that Lis1 and dynactin are differentially involved in different mitotic dynein functions. How does dynein-Golgin160 activity cross-talk with other motors, i.e., kinesins and myosin (Barlan et al., 2013)? Knock-down of the kinesin KIF5B results in longer run lengths of ER-to-Golgi membrane carriers, indicating a negative regulation of ER-to Golgi transport by plus-end motors, and the presence of motors of opposite polarity on ER-to-Golgi cargo (Brown et al., 2014). A variety of scaffolds such as huntingtin have been implicated in the coordination of minus- and plus-end motors for specific retrograde and anterograde axonal transport cargo's (Maday et al., 2014). How is dynein-dynactin-Golgin160 motoring integrated with the microtubule organization around the Golgi? This question is particularly relevant in developing and adult neurons with their complex Golgi morphologies and microtubule organization (Horton et al., 2005; Kuijpers and Hoogenraad, 2011; Kapitein and Hoogenraad, 2015).

The importance of proper dynein function for the Golgi apparatus in neurons is illustrated by genetic disorders that combine mutations in dynein subunits and regulatory factors, with nervous system abnormalities and abnormalities in the Golgi apparatus. In addition, some evidence indicates that dynein malfunction in the Golgi apparatus is an early feature in neurodegenerative disorders such as Amyotrophic lateral sclerosis (van Dis et al., 2014) and Alzheimer's disease (Joshi and Wang, 2015). We have recently found that dysregulation of dynein/dynactin function by BICD2-N overexpression increases Golgi fragmentation in SOD1-ALS mice (van Dis et al., 2014). Knowledge about the role of dynein in and around the Golgi will contribute to our understanding of these disorders.

## Author contributions

DJ wrote the manuscript and made the figures; CH wrote and edited the manuscript.

## Funding

This work was supported by the Internationale Stichting Alzheimer Onderzoek (ISAO #14540, DJ), the Netherlands Organization for Scientific Research (NWO-ALW-VICI, CH), the Foundation for Fundamental Research on Matter ((FOM) CH)), which is part of the NWO, the Netherlands Organization for Health Research and Development (ZonMW-TOP, CH).

### Conflict of interest statement

The authors declare that the research was conducted in the absence of any commercial or financial relationships that could be construed as a potential conflict of interest.

## References

[B1] Akhmanova A. Hammer J. A. III. (2010). Linking molecular motors to membrane cargo. Curr. Opin. Cell Biol. 22, 479–487. 10.1016/j.ceb.2010.04.008 20466533 PMC3393125

[B2] Allan V. J. (2011). Cytoplasmic dynein. Biochem. Soc. Trans. 39, 1169–1178. 10.1042/BST0391169 21936784

[B3] Altan-Bonnet N. Sougrat R. Lippincott-Schwartz J. (2004). Molecular basis for Golgi maintenance and biogenesis. Curr.Opin. Cell Biol. 16, 364–372. 10.1016/j.ceb.2004.06.011 15261668

[B4] Arasaki K. Uemura T. Tani K. Tagaya M. (2007). Correlation of Golgi localization of ZW10 and centrosomal accumulation of dynactin. Biochem. Biophys. Res. Commun. 359, 811–816. 10.1016/j.bbrc.2007.05.188 17560939

[B5] Armbrust K. R. Wang X. Hathorn T. J. Cramer S. W. Chen G. Zu T.. (2014). Mutant beta-III spectrin causes mGluR1alpha mislocalization and functional deficits in a mouse model of spinocerebellar ataxia type 5. J. Neurosci. 34, 9891–9904. 10.1523/JNEUROSCI.0876-14.2014 25057192 PMC4107406

[B6] Arthur A. L. Yang S. Z. Abellaneda A. M, Wildonger, J. (2015). Dendrite arborization requires the dynein cofactor NudE. J. Cell Sci. 128, 2191–2201. 10.1242/jcs.170316 25908857 PMC4450295

[B7] Bacia K. Futai E. Prinz S. Meister A. Daum S. Glatte D.. (2011). Multibudded tubules formed by COPII on artificial liposomes. Sci. Rep. 1:17. 10.1038/srep00017 22355536 PMC3216505

[B8] Banks G. T. Haas M. A. Line S. Shepherd H. L. Alqatari M. Stewart S.. (2011). Behavioral and other phenotypes in a cytoplasmic Dynein light intermediate chain 1 mutant mouse. J. Neurosci. 31, 5483–5494. 10.1523/JNEUROSCI.5244-10.2011 21471385 PMC3096546

[B9] Barlan K. Rossow M. J. Gelfand V. I. (2013). The journey of the organelle: teamwork and regulation in intracellular transport. Curr. Opin. Biol. 25, 483–488. 10.1016/j.ceb.2013.02.018 23510681 PMC3723706

[B10] Brown A. K. Hunt S. D. Stephens D. J. (2014). Opposing microtubule motors control motility, morphology and cargo segregation during ER-to-Golgi transport. Biol. Open 3, 307–313. 10.1242/bio.20147633 24705013 PMC4021352

[B11] Carter A. P. (2013). Crystal clear insights into how the dynein motor moves. J. Cell Sci. 126(Pt 3), 705–713. 10.1242/jcs.120725 23525020

[B12] Clarkson Y. L. Perkins E. M. Cairncross C. J. Lyndon A. R. Skehel P. A. Jackson M. (2014). beta-III spectrin underpins ankyrin R function in Purkinje cell dendritic trees: protein complex critical for sodium channel activity is impaired by SCA5-associated mutations. Hum. Mol. Genet. 23, 3875–3882. 10.1093/hmg/ddu103 24603075 PMC4065159

[B13] Cole N. B. Sciaky N. Marotta A. Song J. Lippincott-Schwartz J. (1996).Golgi dispersal during microtubule disruption: regeneration of Golgi stacks at peripheral endoplasmic reticulum exit sites. Mol. Biol. Cell 7, 631–650. 10.1091/mbc.7.4.631 8730104 PMC275914

[B14] Fiorillo C. Moro F. Yi J. Weil S. Brisca G. Astrea G.. (2014). Novel dynein DYNC1H1 neck and motor domain mutations link distal spinal muscular atrophy and abnormal cortical development. Hum. Mutat. 35, 298–302. 10.1002/humu.22491 24307404 PMC4109683

[B15] Fu M. M. Holzbaur E. L. (2014). Integrated regulation of motor-driven organelle transport by scaffolding proteins. Trends Cell Biol. 24, 564–574. 10.1016/j.tcb.2014.05.002 24953741 PMC4177981

[B16] Gardiol A. Racca C. Triller A. (1999). Dendritic and postsynaptic protein synthetic machinery. J. Neurosci. 19, 168–179. 9870948 10.1523/JNEUROSCI.19-01-00168.1999 PMC6782360

[B17] Ha K. D. Clarke B. A. Brown W. J. (2012). Regulation of the Golgi complex by phospholipid remodeling enzymes. Biochim. Biophys. Acta 1821, 1078–1088. 10.1016/j.bbalip.2012.04.004 22562055 PMC3399269

[B18] Hafezparast M. Klocke R. Ruhrberg C. Marquardt A. Ahmad-Annuar A. Bowen S.. (2003). Mutations in dynein link motor neuron degeneration to defects in retrograde transport. Science 300, 808–812. 10.1126/science.1083129 12730604

[B19] Hanus C. Schuman E. M. (2013). Proteostasis in complex dendrites. Nat. Rev. Neurosci. 14, 638–648. 10.1038/nrn3546 23900412

[B20] Harada A. Takei Y. Kanai Y. Tanaka Y. Nonaka S. Hirokawa N. (1998). Golgi vesiculation and lysosome dispersion in cells lacking cytoplasmic dynein. J. Cell Biol. 141, 51–59. 10.1083/jcb.141.1.51 9531547 PMC2132725

[B21] Hoogenraad C. C. Akhmanova A. Howell S. A. Dortland B. R. De Zeeuw C. I. Willemsen R.. (2001). Mammalian Golgi-associated Bicaudal-D2 functions in the dynein-dynactin pathway by interacting with these complexes. EMBO J. 20, 4041–4054. 10.1093/emboj/20.15.4041 11483508 PMC149157

[B22] Hoogenraad C. C. Wulf P. Schiefermeier N. Stepanova T. Galjart N. Small J. V.. (2003). Bicaudal D induces selective dynein-mediated microtubule minus end-directed transport. EMBO J. 22, 6004–6015. 10.1093/emboj/cdg592 14609947 PMC275447

[B23] Horton A. C. Rácz B. Monson E. E. Lin A. L. Weinberg R. J. Ehlers M. D. (2005). Polarized secretory trafficking directs cargo for asymmetric dendrite growth and morphogenesis. Neuron 48, 757–771. 10.1016/j.neuron.2005.11.005 16337914

[B24] Jaarsma D. van den Berg R. Wulf P. S. van Erp S. Keijzer N. Schlager M. A.. (2014). A role for Bicaudal-D2 in radial cerebellar granule cell migration. Nat. Commun. 5, 3411. 10.1038/ncomms4411 24614806

[B25] Jha R. Surrey T. (2015). Regulation of processive motion and microtubule localization of cytoplasmic dynein. Biochem. Soc. Trans. 43, 48–57. 10.1042/BST20140252 25619245

[B26] Joshi G. Wang Y. (2015). Golgi defects enhance APP amyloidogenic processing in Alzheimer's disease. Bioessays 37, 240–247. 10.1002/bies.201400116 25546412 PMC4407201

[B27] Kapitein L. C. Hoogenraad C. C. (2015). Building the neuronal microtubule cytoskeleton. Neuron 87, 492–506. 10.1016/j.neuron.2015.05.046 26247859

[B28] Klumperman J. (2011). Architecture of the mammalian Golgi. Cold Spring Harb. Perspect. Biol. 3. 10.1101/cshperspect.a005181 21502307 PMC3119909

[B29] Kondylis V. Rabouille C. (2009). The Golgi apparatus: lessons from Drosophila. FEBS Lett. 583, 3827–3838. 10.1016/j.febslet.2009.09.048 19800333

[B30] Kong S. Du X. Peng C. Wu Y. Li H. Jin X.. (2013). Dlic1 deficiency impairs ciliogenesis of photoreceptors by destabilizing dynein. Cell Res 23, 835–850. 10.1038/cr.2013.59 23628724 PMC3674393

[B31] Kuijpers M. Hoogenraad C. C. (2011). Centrosomes, microtubules and neuronal development. Mol. Cell. Neurosci. 48, 349–358. 10.1016/j.mcn.2011.05.004 21722732

[B32] Lam C. Vergnolle M. A. Thorpe L. Woodman P. G. Allan V. J. (2010). Functional interplay between LIS1, NDE1 and NDEL1 in dynein-dependent organelle positioning. J. Cell Sci. 123(Pt 2), 202–212. 10.1242/jcs.059337 20048338

[B33] Levy J. R. Sumner C. J. Caviston J. P. Tokito M. K. Ranganathan S. Ligon L. A.. (2006). A motor neuron disease-associated mutation in p150Glued perturbs dynactin function and induces protein aggregation. J. Cell Biol. 172, 733–745. 10.1083/jcb.200511068 16505168 PMC2063705

[B34] Lin C. H. Li H. Lee Y. N. Cheng Y. J. Wu R. M. Chien C. T. (2015). Lrrk regulates the dynamic profile of dendritic Golgi outposts through the golgin Lava lamp. J. Cell Biol. 210, 471–483. 10.1083/jcb.201411033 26216903 PMC4523617

[B35] Lipka J. Kuijpers M. Jaworski J. Hoogenraad C. C. (2013). Mutations in cytoplasmic dynein and its regulators cause malformations of cortical development and neurodegenerative diseases. Biochem. Soc. Trans. 41, 1605–1612. 10.1042/BST20130188 24256262

[B36] Liu Y. Salter H. K. Holding A. N. Johnson C. M. Stephens E. Lukavsky P. J.. (2013). Bicaudal-D uses a parallel, homodimeric coiled coil with heterotypic registry to coordinate recruitment of cargos to dynein. Genes Dev. 27, 1233–1246. 10.1101/gad.212381.112 23723415 PMC3690397

[B37] Lloyd T. E. Machamer J. O'Hara K. Kim J. H. Collins S. E. Wong M. Y.. (2012). The p150(Glued) CAP-Gly domain regulates initiation of retrograde transport at synaptic termini. Neuron 74, 344–360. 10.1016/j.neuron.2012.02.026 22542187 PMC3353876

[B38] Lord C. Ferro-Novick S. Miller E. A. (2013). The highly conserved COPII coat complex sorts cargo from the endoplasmic reticulum and targets it to the golgi. Cold Spring Harb. Perspect. Biol. 5. 10.1101/cshperspect.a013367 23378591 PMC3552504

[B39] Lu L. Hong W. (2014). From endosomes to the trans-Golgi network. Semin. Cell Dev. Biol. 31, 30–39. 10.1016/j.semcdb.2014.04.024 24769370

[B40] Maday S. Twelvetrees A. E. Moughamian A. J. Holzbaur E. L. (2014). Axonal transport: cargo-specific mechanisms of motility and regulation. Neuron 84, 292–309. 10.1016/j.neuron.2014.10.019 25374356 PMC4269290

[B41] Majeed W. Liu S. Storrie B. (2014). Distinct sets of Rab6 effectors contribute to ZW10–and COG-dependent Golgi homeostasis. Traffic 15, 630–647. 10.1111/tra.12167 24575842 PMC4016170

[B42] McKenney R. J. Huynh W. Tanenbaum M. E. Bhabha G. Vale R. D. (2014). Activation of cytoplasmic dynein motility by dynactin-cargo adapter complexes. Science 345, 337–341. 10.1126/science.1254198 25035494 PMC4224444

[B43] Moughamian A. J. Osborn G. E. Lazarus J. E. Maday S. Holzbaur E. L. (2013). Ordered recruitment of dynactin to the microtubule plus-end is required for efficient initiation of retrograde axonal transport. J. Neurosci. 33, 13190–13203. 10.1523/JNEUROSCI.0935-13.2013 23926272 PMC3735891

[B44] Munro S. (2011). The golgin coiled-coil proteins of the Golgi apparatus. Cold Spring Harb. Perspect. Biol. 3. 10.1101/cshperspect.a005256 21436057 PMC3098672

[B45] Nakamura N. Wei J. H. Seemann J. (2012).Modular organization of the mammalian Golgi apparatus. Curr. Opin. Cell Biol. 24, 467–474. 10.1016/j.ceb.2012.05.009 22726585 PMC3495003

[B46] Neveling K. Martinez-Carrera L. A. Hölker I. Heister A. Verrips A. Hosseini-Barkooie S. M.. (2013). Mutations in BICD2, which encodes a Golgin and important motor adaptor, cause congenital autosomal-dominant spinal muscular atrophy. Am. J. Hum. Genet. 92, 946–954. 10.1016/j.ajhg.2013.04.011 23664116 PMC3675237

[B47] Niu Y. Zhang C. Sun Z. Hong Z. Li K. Sun D.. (2013). PtdIns(4)P regulates retromer-motor interaction to facilitate dynein-cargo dissociation at the trans-Golgi network. Nat. Cell Biol. 15, 417–429. 10.1038/ncb2710 23524952

[B48] Ori-McKenney K. M. Jan L. Y. Jan Y. N. (2012). Golgi outposts shape dendrite morphology by functioning as sites of acentrosomal microtubule nucleation in neurons. Neuron 76, 921–930. 10.1016/j.neuron.2012.10.008 23217741 PMC3523279

[B49] Palmer K. J. Hughes H. Stephens D. J. (2009). Specificity of cytoplasmic dynein subunits in discrete membrane-trafficking steps. Mol.Biol. Cell 20, 2885–2899. 10.1091/mbc.E08-12-1160 19386764 PMC2695796

[B50] Pandey J. P. Smith D. S. (2011). A Cdk5-dependent switch regulates Lis1/Ndel1/dynein-driven organelle transport in adult axons. J. Neurosci. 31, 17207–17219. 10.1523/JNEUROSCI.4108-11.2011 22114287 PMC3249231

[B51] Papoulas O. Hays T. S. Sisson J. C. (2005). The golgin Lava lamp mediates dynein-based Golgi movements during Drosophila cellularization. Nat. Cell Biol. 7, 612–618. 10.1038/ncb1264 15908943

[B52] Peeters K. Bervoets S. Chamova T. Litvinenko I. De Vriendt E. Bichev S.. (2015). Novel mutations in the DYNC1H1 tail domain refine the genetic and clinical spectrum of dyneinopathies. Hum. Mutat. 36, 287–291. 10.1002/humu.22744 25512093

[B53] Peeters K. Litvinenko I. Asselbergh B. Almeida-Souza L. Chamova T. Geuens T.. (2013). Molecular defects in the motor adaptor BICD2 cause proximal spinal muscular atrophy with autosomal-dominant inheritance. Am. J. Hum. Genet. 92, 955–964. 10.1016/j.ajhg.2013.04.013 23664119 PMC3675262

[B54] Pfister K. K. (2015). Distinct functional roles of cytoplasmic dynein defined by the intermediate chain isoforms. Exp. Cell Res. 334, 54–60. 10.1016/j.yexcr.2014.12.013 25576383 PMC4433767

[B55] Pfister K. K. Shah P. R. Hummerich H. Russ A. Cotton J. Annuar A. A.. (2006). Genetic analysis of the cytoplasmic dynein subunit families. PLoS Genet. 2:e1. 10.1371/journal.pgen.0020001 16440056 PMC1331979

[B56] Quassollo G. Wojnacki J. Salas D. A. Gastaldi L. Marzolo M. P. Conde C.. (2015). A RhoA signaling pathway regulates dendritic golgi outpost formation. Curr. Biol. 25, 971–982. 10.1016/j.cub.2015.01.075 25802147

[B57] Raaijmakers J. A. Tanenbaum M. E. Medema R. H. (2013). Systematic dissection of dynein regulators in mitosis. J. Cell Biol. 201, 201–215. 10.1083/jcb.201208098 23589491 PMC3628524

[B58] Rios R. M. (2014). The centrosome-Golgi apparatus nexus. Philos. Trans. R. Soc. Lond. B Biol. Sci 369. 10.1098/rstb.2013.0462 25047616 PMC4113106

[B59] Roberts A. J. Kon T. Knight P. J. Sutoh K. Burgess S. A. (2013). Functions and mechanics of dynein motor proteins. Nat. Rev. Mol. Cell Biol. 14, 713–726. 10.1038/nrm3667 24064538 PMC3972880

[B60] Salcedo-Sicilia L. Granell S. Jovic M. Sicart A. Mato E. Johannes L.. (2013). betaIII spectrin regulates the structural integrity and the secretory protein transport of the Golgi complex. J. Biol. Chem. 288, 2157–2166. 10.1074/jbc.M112.406462 23233669 PMC3554888

[B61] Sasaki S. Mori D. Toyo-oka K. Chen A. Garrett-Beal L. Muramatsu M.. (2005). Complete loss of Ndel1 results in neuronal migration defects and early embryonic lethality. Mol. Cell. Biol. 25, 7812–7827. 10.1128/MCB.25.17.7812-7827.2005 16107726 PMC1190282

[B62] Schlager M. A. Hoang H. T. Urnavicius L. Bullock S. L. Carter A. P. (2014a). *In vitro* reconstitution of a highly processive recombinant human dynein complex. Embo J. 33, 1855–1868. 10.15252/embj.201488792 24986880 PMC4158905

[B63] Schlager M. A. Serra-Marques A. Grigoriev I. Gumy L. F. Esteves da Silva M. Wulf P. S.. (2014b). Bicaudal d family adaptor proteins control the velocity of Dynein-based movements. Cell Rep. 8, 1248–1256. 10.1016/j.celrep.2014.07.052 25176647

[B64] Schroeder C. M. Ostrem J. M. Hertz N. T. Vale R. D. (2014). A Ras-like domain in the light intermediate chain bridges the dynein motor to a cargo-binding region. Elife 3. 10.7554/elife.03351 25272277 PMC4359372

[B65] Scoto M. Rossor A. M. Harms M. B. Cirak S. Calissano M. Robb S.. (2015). Novel mutations expand the clinical spectrum of DYNC1H1-associated spinal muscular atrophy. Neurology 84, 668–679. 10.1212/WNL.0000000000001269 25609763 PMC4336105

[B66] Splinter D. Razafsky D. S. Schlager M. A. Serra-Marques A. Grigoriev I. Demmers J.. (2012). BICD2, dynactin, and LIS1 cooperate in regulating dynein recruitment to cellular structures. Mol. Biol. Cell 23, 4226–4241. 10.1091/mbc.E12-03-0210 22956769 PMC3484101

[B67] Stephens D. J. (2012). Functional coupling of microtubules to membranes - implications for membrane structure and dynamics. J. Cell Sci. 125, 2795–2804. 10.1242/jcs.097675 22736043

[B68] Tacik P. Fiesel F. C. Fujioka S. Ross O. A. Pretelt F. Castañeda Cardona C.. (2014). Three families with Perry syndrome from distinct parts of the world. Parkinsonism Relat. Disord. 20, 884–888. 10.1016/j.parkreldis.2014.05.004 24881494 PMC4125456

[B69] Tan S. C. Scherer J. Vallee R. B. (2011). Recruitment of dynein to late endosomes and lysosomes through light intermediate chains. Mol. Biol. Cell 22, 467–477. 10.1091/mbc.E10-02-0129 21169557 PMC3038645

[B70] Teuling E. van Dis V. Wulf P. S. Haasdijk E. D. Akhmanova A. Hoogenraad C. C.. (2008). A novel mouse model with impaired dynein/dynactin function develops amyotrophic lateral sclerosis (ALS)-like features in motor neurons and improves lifespan in SOD1-ALS mice. Hum. Mol. Genet. 17, 2849–2862. 10.1093/hmg/ddn182 18579581

[B71] Torisawa T. Ichikawa M. Furuta A. Saito K. Oiwa K. Kojima H.. (2014). Autoinhibition and cooperative activation mechanisms of cytoplasmic dynein. Nat. Cell Biol. 16, 1118–1124. 10.1038/ncb3048 25266423

[B72] Torisawa T. Nakayama A. Furuta K. Yamada M. Hirotsune S. Toyoshima Y. Y. (2011). Functional dissection of LIS1 and NDEL1 towards understanding the molecular mechanisms of cytoplasmic dynein regulation. J. Biol. Chem. 286, 1959–1965. 10.1074/jbc.M110.169847 21036906 PMC3023492

[B73] Toropova K. Zou S. Roberts A. J. Redwine W. B. Goodman B. S. Reck-Peterson S. L.. (2014). Lis1 regulates dynein by sterically blocking its mechanochemical cycle. eLife 3. 10.7554/elife.03372 25380312 PMC4359366

[B74] Urnavicius L. Zhang K. Diamant A. G. Motz C. Schlager M. A. Yu M.. (2015). The structure of the dynactin complex and its interaction with dynein. Science 347, 1441–1446. 10.1126/science.aaa4080 25814576 PMC4413427

[B75] Vallee R. B. McKenney R. J. Ori-McKenney K. M. (2012). Multiple modes of cytoplasmic dynein regulation. Nat. Cell Biol. 14, 224–230. 10.1038/ncb2420 22373868

[B76] van Dis V. Kuijpers M. Haasdijk E. D. Teuling E. Oakes S. A. Hoogenraad C. C.. (2014). Golgi fragmentation precedes neuromuscular denervation and is associated with endosome abnormalities in SOD1-ALS mouse motor neurons. Acta Neuropathol. Commun. 2:38. 10.1186/2051-5960-2-38 24708899 PMC4023628

[B77] Varma D. Dujardin D. L. Stehman S. A. Vallee R. B. (2006). Role of the kinetochore/cell cycle checkpoint protein ZW10 in interphase cytoplasmic dynein function. J. Cell Biol. 172, 655–662. 10.1083/jcb.200510120 16505164 PMC2063698

[B78] Varma D. Monzo P. Stehman S. A. Vallee R. B. (2008). Direct role of dynein motor in stable kinetochore-microtubule attachment, orientation, and alignment. J. Cell Biol. 182, 1045–1054. 10.1083/jcb.200710106 18809721 PMC2542467

[B79] Walenta J. H. Didier A. J. Liu X. Krämer H. (2001). The Golgi-associated hook3 protein is a member of a novel family of microtubule-binding proteins. J. Cell Biol. 152, 923–934. 10.1083/jcb.152.5.923 11238449 PMC2198811

[B80] Wassmer T. Attar N. Harterink M. van Weering J. R. Traer C. J. Oakley J.. (2009). The retromer coat complex coordinates endosomal sorting and dynein-mediated transport, with carrier recognition by the trans-Golgi network. Dev. Cell 17, 110–122. 10.1016/j.devcel.2009.04.016 19619496 PMC2714578

[B81] Watanabe T. Bochimoto H. Koga D. Hosaka M. Ushiki T. (2014). Functional implications of the Golgi and microtubular network in gonadotropes. Mol. Cell Endocrinol. 385, 88–96. 10.1016/j.mce.2013.10.003 24121198

[B82] Watson P. Forster R. Palmer K. J. Pepperkok R. Stephens D. J. (2005). Coupling of ER exit to microtubules through direct interaction of COPII with dynactin. Nat. Cell Biol. 7, 48–55. 10.1038/ncb1206 15580264 PMC1592520

[B83] Yadav S. Linstedt A. D. (2011). Golgi positioning. Cold Spring Harb. Perspect. Biol. 3. 10.1101/cshperspect.a005322 21504874 PMC3101843

[B84] Yadav S. Puthenveedu M. A. Linstedt A. D. (2012). Golgin160 recruits the dynein motor to position the Golgi apparatus. Dev. Cell 23, 153–165. 10.1016/j.devcel.2012.05.023 22814606 PMC3417773

[B85] Ye B. Zhang Y. Song W. Younger S. H. Jan L. Y. Jan Y. N. (2007). Growing dendrites and axons differ in their reliance on the secretory pathway. Cell 130, 717–729. 10.1016/j.cell.2007.06.032 17719548 PMC2020851

[B86] Yi J. Y. Ori-McKenney K. M. McKenney R. J. Vershinin M. Gross S. P. Vallee R. B. (2011). High-resolution imaging reveals indirect coordination of opposite motors and a role for LIS1 in high-load axonal transport. J. Cell Biol. 195, 193–201. 10.1083/jcb.201104076 22006948 PMC3198168

[B87] Youn Y. H. Pramparo T. Hirotsune S. Wynshaw-Boris A. (2009). Distinct dose-dependent cortical neuronal migration and neurite extension defects in Lis1 and Ndel1 mutant mice. J. Neurosci. 29, 15520–15530. 10.1523/JNEUROSCI.4630-09.2009 20007476 PMC2824645

[B88] Zheng Y. Wildonger J. Ye B. Zhang Y. Kita A. Younger S. H.. (2008). Dynein is required for polarized dendritic transport and uniform microtubule orientation in axons. Nat. Cell Biol. 10, 1172–1180. 10.1038/ncb1777 18758451 PMC2588425

[B89] Zhu X. Kaverina I. (2013). Golgi as an MTOC: making microtubules for its own good. Histochem. Cell Biol. 140, 361–367. 10.1007/s00418-013-1119-4 23821162 PMC3748218

